# Place of Death for Israeli Cancer Patients Over a 20-Year Period: Reducing Hospital Deaths, but Barriers Remain

**DOI:** 10.1093/oncolo/oyad141

**Published:** 2023-06-01

**Authors:** Yuval Shalev Many, Pesach Shvartzman, Ido Wolf, Barbara G Silverman

**Affiliations:** Sackler School of Medicine, Tel Aviv University, Tel Aviv, Israel; Pain and Palliative Care Unit, Department of Family Medicine, Ben Gurion University, Beer Sheva, Israel; Sackler School of Medicine, Tel Aviv University, Tel Aviv, Israel; The Oncology Division, Tel Aviv Sourasky Medical Center, Tel Aviv, Israel; Sackler School of Medicine, Tel Aviv University, Tel Aviv, Israel; Israel National Cancer Registry, Israel Ministry of Health, Ramat Gan, Israel

**Keywords:** home hospice care, dying at home, patients with cancer, end of life care, health care disparities

## Abstract

**Background:**

Cancer remains a leading cause of mortality worldwide. While the main focus of palliative care (PC) is quality of life, the elements that comprise the quality of death are often overlooked. Dying at home, with home-hospice-care (HHC) support, rather than in-hospital, may increase patient satisfaction and decrease the use of invasive measures. We examined clinical and demographic characteristics associated with out-of-hospital death among patients with cancer, which serves as a proxy measure for HHC deaths.

**Methods:**

Using death certification data from the Israel Central Bureau of Statistics, we analyzed 209,158 cancer deaths between 1998 and 2018 in Israel including demographic information, cause of death, and place of death (POD). A multiple logistic regression model was constructed to identify factors associated with out-of-hospital cancer deaths.

**Results:**

Between 1998 and 2018, 69.1% of cancer deaths occurred in-hospital, and 30.8% out-of-hospital. Out-of-hospital deaths increased by 1% annually during the study period. Older patients and those dying of solid malignancies were more likely to die out-of-hospital (OR = 2.65, OR = 1.93, respectively). Likelihood of dying out-of-hospital varied with area of residency; patients living in the Southern district were more likely than those in the Jerusalem district to die out-of-hospital (OR = 2.37).

**Conclusion:**

The proportion of cancer deaths occurring out-of-hospital increased during the study period. We identified clinical and demographic factors associated with POD. Differences between geographical areas probably stem from disparity in the distribution of PC services and highlight the need for increasing access to primary EOL care. However, differences in age and tumor type probably reflect cultural changes and suggest focusing on educating patients, families, and physicians on the benefits of PC.

## Teaser

Spending the last days of life at home has been shown to be associated with less invasive measures and better quality of life at such a critical time. This study identified factors associated with increased likelihood of home death among cancer patients.

**Implications for Practice**

This study identified factors associated with increased likelihood of home death among cancer patients. Spending the last days of life at home has been shown to be associated with less invasive measures and better quality of life at such a critical time. Our findings provide valuable tools for both practicing physicians and the health care authorities, as they point to unique populations that show reduced likelihood of dying at home. Addressing specific needs of these populations, including expanding access to PC services, and educating patients, families, and physicians, could improve EOL care for cancer patients.

## Introduction

Cancer is a leading cause of death (COD) worldwide,^1,2^ and there is a massive focus on patient choices as they enter the terminal stage of illness. One of the crucial decisions for patients is where they prefer to spend their last days, and many studies show that the most common preference is to die at home.^3-5^ Moreover, home-hospice-care (HHC) that focuses on care and quality of end-of-life (EOL) may increase patient satisfaction and decrease hospital admissions.^6,7^

A study of place of death (POD) for patients with cancer dying in the US between 1999 and 2015 (*n* = 9 646 498), found that young people (aged 0-44), Black and Asian Americans, and those of Hispanic origin were at increased likelihood of dying in hospital, while people who were married or widowed were the most likely to die at home.^8^ A comparison of cancer deaths in 14 countries found wide variations in the proportion of patients dying at home, with the highest in Mexico (57%) and the lowest rate in Korea (12%). The differences in the proportion of cancer deaths occurring at home could be partly explained by differences in availability and accessibility to hospitals, long-term care beds, general practitioners, and cultural differences.^9^

A study published in 2021 on cancer deaths in Israel identified several factors associated with deaths at home. Death at home was most frequent among patients diagnosed with brain tumors, and among persons in the fourth to the seventh decades of life. This study was limited to people included in the 1995 Israeli Census diagnosed with cancer from 2008 to 2015.^10^

The Israel National Health Insurance Law, passed in 1995, guarantees universal healthcare, including palliative and hospice services, to all citizens and permanent residents.^11,12^ Israel’s population consists of many different groups, but the largest axis is ethnic origin, between Israeli Jews (74·8%) and Israeli Arabs (20·8%). The Israeli Arabs have traditionally been classified by religion: Muslim (84·5%), Druze (7·9%), and Christian (7·6%). Universal access to health care minimizes the financial burden on patients but disparities persist, based on socioeconomic factors and geographic distribution.^11^

In 2009, the Israeli Ministry of Health (MOH) established a policy set to implement palliative care (PC) guidelines.^13^ During the last decade, implementation programs for PC services and HHC were developed and improved.^13-16^ Currently, there are 9.6 million residents in Israel, and PC is provided by 11 HHC programs and 2 hospital-based hospices with a total of 44 beds. On average, there are 1.1 hospital beds in hospices for every 100 000 residents.^17^

Comprehensive data are required to quantify the current need for HHC in Israel and assess the extent to which this need is being met. In this study, we performed an analysis of person-level de-identified death certification data in order to identify the factors associated with out-of-hospital deaths among cancer patients.

## Methods

The study is a retrospective cohort study conducted at Tel Aviv University. Data were obtained from the “Timna” system, a national research platform established by the Israel MOH that provides access to de-identified health services and demographic data for the purpose of health services research. We received access to the data abstracted by the Israel Central Bureau of Statistics (CBS) from certifications of deaths of citizens and permanent residents who died in Israel between 1998 and 2018 and for whom the primary COD, as recorded on the death certification, was a malignant disease.

### Measures

The CBS death data set included the following variables: date of birth, date of death, COD coded according to the International Classification of Disease, Version 10 (ICD10), setting of death (coded as at home, in hospital, abroad, other), sex, and district of residence at the time of death (Jerusalem, Northern, Haifa, Central, Tel Aviv, Southern). No information was available on the population group of the deceased (Jewish, Arab, Other). We derived the following additional variables:

#### Place of death:

A dichotomous variable (in-hospital/out-of-hospital) derived from the “setting of death” variable was used as a surrogate measure of HHC death. This variable was created from the “Place of Death” variable, found in the CBS database. For the purposes of this analysis, we classified deaths occurring at home, abroad, or in other locations as “out of hospital.”

#### Cause of death:

A categorical variable derived from the ICD10 code for the primary COD recorded on the death notification. The causes of death were divided into 2 categories, solid and hematologic malignancies. The category of solid malignancies was further divided into cancer subgroups (pancreas, prostate, breast, colorectal, lung, unknown-primary, and other) (Supplementary Table. S1).^2^

#### Age group at time of death:

Calculated as the difference between date of birth and date of death, and stratified into the categories 0-44, 45-54, 55-64, 65-74, 75, and over.

### Statistical Analysis

Descriptive statistics for all the variables defined as categorical or dichotomous are presented as mode and frequency distribution. Univariate analyses were performed using the Chi-square test to test the association of gender, age group, cancer subgroups, and district with POD (in-hospital vs. out-of-hospital). A *P*-value of .05 was considered significant in the descriptive analysis, as a means of identifying factors to be included in a multivariate analysis. Based on the results of the univariate analysis, we performed a multivariate logistic regression analysis using death out-of-hospital as the dependent dichotomous variable. These analyses were performed using SPSS statistical software (version 25). In addition, we used the U.S. National Cancer Institute’s Joinpoint Regression Analysis program, version 4.9.1.0, to calculate annual percent changes (APCs) and 95% confidence intervals (CIs) in order to quantify trends over time in the proportion of deaths occurring out-of-hospital, by demographic characteristics and cancer types during the study period, using *T*-tests to determine whether APCs were significant.^18^

## Results

### Patient Characteristics

Between 1998 and 2018, there were 209,158 deaths due to cancer in Israel. While 69.1% of deaths occurred in hospitals, 30.8% were out of hospital. Results of the analysis of POD, by demographic characteristics and cancer type, are displayed in Table 1 and Supplementary Table S2. The proportion of out-of-hospital deaths was similar among persons 0-20 and 21-44 years of age (16.0%, 95% CI 14.1-17.9, and 17.7%, 95% CI 16.8-18.5, respectively). For this reason, these 2 age groups were combined for analysis. Almost half of recorded cancer deaths occurred in persons 75 and over, and the proportion of out-of-hospital death increased sharply by age (17.4% of deaths among people ages 44 and under, 22.1% for ages 45-54, 25.5% for ages 65-74, and 36% for ages 75 and over). Overall, women were more likely to die out-of-hospital than men (Supplementary Fig. S1). Compared to those who had hematologic malignancies, patients who had solid tumors were more likely to die out-of-hospital (32.5% and 20.1%, respectively).

**Table 1.** Independent variables and types of cancer deaths in Israel 1998 to2018.

	All	In hospital	Out of hospital	*P*-value^*^
Age groups				<.001
0-44	4.3%	82.6%	17.4%	
45-54	7.1%	77.9%	22.1%	
55-64	15.1%	74.5%	25.5%	
65-74	23.9%	71.4%	28.6%	
Above 75	49.6%	64.0%	36.0%	
Sex				<.001
Male	50.3%	70.6%	29.4%	
Female	49.7%	67.6%	32.4%	
District of residence				<.001
Jerusalem	8.6%	78.0%	22.0%	
Northern	13.0%	65.6%	34.4%	
Haifa	15.3%	64.0%	36.0%	
Central	23.5%	74.8%	25.2%	
Tel Aviv	24.4%	70.4%	29.6%	
Southern	13.9%	60.4%	39.6%	
Solid/Hematologic				<.001
Solid	87.0%	67.5%	32.5%	
Hematologic	13.0%	79.9%	20.1%	
Cancer Type				<.001
Unknown primary	4.9%	65.2%	34.8%	
Hematologic	13.1%	79.9%	20.1%	
Prostate	4.1%	64.2%	35.8%	
Breast	9.8%	67.5%	32.5%	
Colorectal	12.6%	65.1%	34.9%	
Lung	16.0%	71.5%	28.5%	
Pancreas	7.5%	66.7%	33.3%	
Other	31.5%	67.5%	32.4%	

^*^Calculated using chi-square exact test for “age group” using linear by linear.

A *P*-value of < .5 was considered significant.

### Trends over time in out-of-hospital deaths, Joinpoint analysis

**Figure 1.**
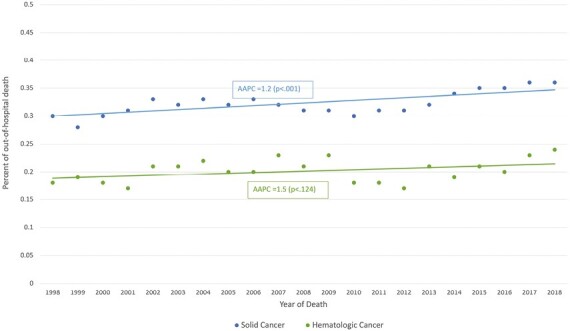
Proportion of deaths occurring out of hospital, by category (solid or hematologic malignancies), Israel, 1998-2018* .*Solid lines represent the average annual percent change (AAPC) throughout the study.

Of deaths due to solid malignancies, 36% occurred out-of-hospital in 2018 compared to 29% in 1998, a 1.2% rise per year (average annual percent change (AAPC) = +1.2%, *P*-value = <.001). Of deaths due to hematologic malignancies, 24% occurred out of hospital in 2018, compared to 18% in 1998, a non-significant increase (AAPC = +1.5%, *P*-value = .124) (Fig. 1, Supplementary Table S3).

**Figure 2.**
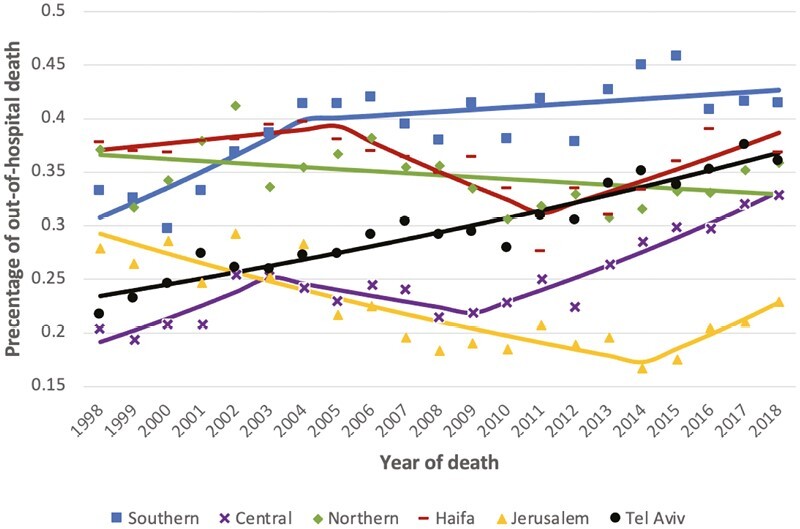
Out-of-hospital cancer deaths by district of resident, Israel, 1998-2018*. *Solid lines represent the study period’s annual percent change (APC).

Trends over time varied by district. We observed significant increases in the proportion of deaths occurring out-of-hospital in the Southern, Central, Tel-Aviv and Haifa districts, while in Jerusalem and the Northern districts we saw a decrease over the study period. In the Southern district, there was a significant increase of 4.4% per year between 1998 and 2004 (APC = +4.4, *P*-value = .02), and another non-significant rise from 2004 to 2018 (APC = +0.5, *P*-value = .207), with 42% of cancer deaths occurring out of hospital in 2018, the highest percentage in Israel. Significant increases were observed in the Tel Aviv district over the study period (APC = +2.3, *P*-value = <.001). In the Northern district, the proportion of out-of-hospital deaths decreased by 1.5% per year in 1998-2018 (APC = −1.5%, *P*-value = .055) (Fig. 2; Supplementary Table S4).

### Multivariate analysis

Multivariate logistic regression confirmed the association of age, gender, district of residence, tumor type and year of death with the likelihood of dying out of hospital (Table 2). The full model included all cancer deaths, stratifying between hematologic and solid tumors, and separate models evaluated the effect of age, gender, district of residence and year of death in persons with solid tumors and hematologic tumors. With each year over the study period, the proportion of deaths occurring out of hospital increased by 1%. The odds ratio of dying out of hospital for patients with cancer aged above 75 was 2.65 compared to the youngest patient category. The proportion of out of hospital deaths in the Southern district was more than twice that in the Jerusalem district (OR = 2.37, 95% CI (2.27, 2.48)). In additions, patients with solid tumors were nearly twice as likely to die out of hospital as those with hematologic cancer (OR = 1.93, 95% CI (1.87, 1.99)).

**Table 2.** Multivariable logistic regression models of the factors associated with out-of-hospital cancer death in Israel 1998-2018.

Subgroup	Odds ratio 95% CI (lower, upper) for out-of-hospital death association		
	All cancer types (*n* = 206,373)	Solid cancer (*n* = 179,204)	Hematologic cancer (*n* = 27,169)
Age groups			
0-44	1.00 (ref.)	1.00 (ref.)	1.00 (ref.)
45-54	1.27 (1.18, 1.36)	1.23 (1.14, 1.32)	1.46 (1.15, 1.87)
55-64	1.54 (1.45, 1.64)	1.49 (1.40, 1.59)	1.80 (1.46, 2.21)
65-74	1.85 (1.74, 1.96)	1.78 (1.67, 1.90)	2.35 (1.95, 2.85)
Above 75	2.65 (2.51, 2.81)	2.49 (2.34, 2.64)	4.36 (3.63, 5.23)
Sex			
Male	0.85 (0.84, 0.87)	0.86 (0.84, 0.87)	0.85 (0.80, 0.91)
Female	1.00 (ref.)	1.00 (ref.)	1.00 (ref.)
District			
Jerusalem	1.00 (ref.)	1.00 (ref.)	1.00 (ref.)
Central	1.19 (1.14, 1.24)	1.19 (1.14, 1.24)	1.18 (1.03, 1.34)
Tel Aviv	1.43 (1.37, 1.49)	1.43 (1.37, 1.50)	1.38 (1.22, 1.57)
Northern	1.94 (1.86, 2.03)	1.94 (1.85, 2.03)	1.98 (1.72, 2.27)
Haifa	1.98 (1.90, 2.07)	2.00 (1.91, 2.09)	1.78 (1.56, 2.04)
Southern	2.37 (2.27, 2.48)	2.44 (2.33, 2.55)	1.84 (1.60, 2.11)
Cancer type			
Hematologic	1.00 (ref.)	—	—
Solid	1.93 (1.87, 1.99)	—	—
Death year	1.01 (1.00, 1.01)	1.01 (1.00, 1.01)	1.00 (1.00, 1.01)

## Discussion

Using data on all deaths due to cancer in the Israeli population between 1998 and 2018, we conducted an analysis of trends in the occurrence of out-of-hospital deaths. We found that among persons dying of cancer, the likelihood of dying out-of-hospital increased over the last 20 years, and is highly dependent on cancer type and demographic factors particularly area of residence. Previous studies have shown that home is the preferred POD both among patients with cancer, and in the general population at large. The ability to choose a preferred POD is regarded as one of the criteria most important for EOL care.^3-5^

Our findings are consistent with prior cross-national research in Europe, which showed that increasing the availability of PC services reduces the rate of in-hospital deaths in persons suffering from terminal illnesses.^19^ In the US, the proportion of deaths occurring at home has increased in recent years, a change that may be explained by increased availability of PC services.^20,21^ The relationship between POD and substantial PC availability was also explored in a recent population-based study in 12 Latin American countries. The authors found that the proportion of in-hospital and home deaths varies significantly between countries, and suggested that factors such as health system resources, policy, and legislation influenced POD.^22^ As in other countries, we found an apparent link between the availability of PC and the frequency of out-of-hospital deaths. The Jerusalem district, which, according to a quality assessment performed by the Israel MOH in 2018 was characterized by limited availability of PC services in the community,^16^ also had the lowest proportion of out-of-hospital cancer deaths of any district for most of the study period. In contrast, the Southern district, with the highest proportion of out-of-hospital deaths, is characterized by rural minority communities, and has a specific HHC policy with unique solutions for their patients, such as the use of dedicated vehicles to traverse the rugged roads for home visits (personal communication, Prof. Pesach Shvartzman Head, Israel Palliative Medicine Association). The Tel Aviv/Central district, which also had high a proportion of out-of-hospital deaths, is characterized by abundant health services which might influence the availability of HHC.^16^ Differences in the distribution of ethnicity and socioeconomic status by district may also have had an impact on our findings.^23,24^

We found that persons with hematologic malignancies were only half as likely as those with solid tumors to die out of hospital, a disparity also evident in other countries.^25^ Treatment course differs between different malignancies. When comparing treatments for hematologic and solid cancer patients in the last 30 days of life, those with hematological malignancies were more likely to receive aggressive care including hospital and intensive care unit (ICU) admissions.^26^ Possible reasons for the differences could be related to the hospice care model which seems to hematologists to be more appropriate for the treatment of solid tumors than for hematologic malignancies,^27^ such as limited access to blood product transfusions.^28-30^ Moreover, hematologists have been shown to be more likely to feel a sense of failure with disease progression compared with oncologists treating primarily solid tumors.^31^ As patients prefer to discuss advance directives and end-of-life planning, including the option for hospice care,^32^ with their oncologists, it is important to expand PC education in specialty oncology and hematology training programs.^33^

Dying out-of-hospital is also associated with increasing age in cancer patients in our study, a trend consistent with previous findings.^34-36^ One prospective study in South Africa compared the stated preferences of cancer patients with actual POD. There were no differences in preferred POD but the mean age of the patients who in fact died at home was 60.2 years, compared to 55 years for those who died at a hospital or facility.^36^ In some countries, there has been a slight increase in the use of PC services, including home care for young adults, teens, and children suffering from terminal cancers^37,38^; however, the proportion of deaths occurring in-hospital in these age groups remains high.^39,40^

In addition to the availability of services and the willingness of physicians to refer patients for PC, another factor of importance is the acceptability of PC to patients and their family members. According to a qualitative systematic review of patient preferences among the general population, people with recent experience of a close friend or relative’s death had a higher preference for HHC.^41^ Furthermore, when families had support from extended family and communicated with the patient about the illness, both the patient and the family preferred treatment at home.^42^ Furthermore, members of the Islamic and Jewish faiths, the 2 predominant religions in Israel, have different approaches toward palliative care which might affect patients’ and family members’ choices.^43^

### Limitations

This study had several limitations. First, the cause of death database did not include patients’ ethnicity, socioeconomic status, or level of religious observance, all of which had been found to have an impact on the POD.^34,35,43^ In addition, the POD variable has only 2 categories (in-hospital and out-of-hospital). People who died out-of-hospital may have died at home or in facilities not classified as general/geriatric hospitals. Moreover, we were unable to take home and inpatient PC capacity at the district level into consideration in the analysis, since these data were not available. An additional source of information bias could be potential COD documentation errors. Deaths of people with a history of cancer who die of other causes may be classified as cancer deaths or the reverse. This work studied cancer deaths during the period from 1998 to 2018, before the outbreak of the COVID-19 global pandemic, which may have had an impact on decisions regarding whether to hospitalize terminally ill patients with cancer.^44^ Despite these limitations, death notification data constitute the most comprehensive information available on POD of patients with end-stage cancer in Israel during the period of the study. As such, they provide a basis for policy recommendations.

In conclusion, despite the fact that the proportion of cancer deaths occurring out-of-hospital has increased in recent years, the majority of patients with cancer continue to pass away within hospital walls. Our research, which analyzed over 200,000 patients with cancer, identified several factors that can affect the likelihood of dying out-of-hospital, including where the patient lives, their age, and the type of cancer they have. To improve EOL care and increase access to high-quality care, there needs to be a focus on improving primary care and home healthcare services, particularly for younger patients and those with hematologic tumors. This includes educating patients, families, and healthcare providers and taking into account religious preferences. In addition, aspects of HHC should be included in programs measuring the quality of community health care.

## Supplementary Material

oyad141_suppl_Supplementary_Tables

oyad141_suppl_Supplementary_Figure_1

## Data Availability

Data were obtained from the “Timna” system in the Israeli Ministry of Health, and includes access to de-identified data abstracted by the Israel Central Bureau of Statistics from certifications. Data will be shared on request to the corresponding author with permission of Israeli MOH.
