# Supplementary material for: Place of Death for Israeli Cancer Patients Over a 20-Year Period: Reducing Hospital Deaths, but Barriers Remain
Source: Oncologist. 2023 Jun 1;28(11):e1092–8. doi: 10.1093/oncolo/oyad141 (PMC10628558; doi:10.1093/oncolo/oyad141)
Supplement: oyad141_suppl_Supplementary_Tables [file oyad141_suppl_supplementary_tables.docx]

**Supplementary Tables**

Table S1. Cause of death divided into categories

| Solid vs. hematologic cancer | | Cancer subgroups | |
| --- | --- | --- | --- |
| Group | ICD10 Codes | Group | ICD10 Codes |
| Solid cancers | C00-C72 | Pancreas | C25 |
| Hematologic cancers | C81-C97 | Prostate | C61 |
|  |  | Breast | C50 |
|  |  | Colorectal | C18-20 |
|  |  | Lung | C34 |
|  |  | Unknown Primary | C80 |
|  |  | Other | C00-C72 excluded C18-20, C25, C34, C50, C61, C80, C81-C97 |

ICD - International Statistical Classification of Diseases

Table S2. Out-of-hospital cancer death by cancer types in Israel 1998-2018

| Cancer category | Out-of-hospital  death | Standard  error | 95% CI (lower, upper) |
| --- | --- | --- | --- |
| Unknown | 34.8% | 0.46 | 33.83, 35.68 |
| Hematologic | 20.1% | 0.24 | 19.66, 20.61 |
| Prostate | 35.8% | 0.51 | 34.75, 36.76 |
| Breast | 32.5% | 0.32 | 31.82, 33.09 |
| Colorectal | 34.9% | 0.29 | 34.57, 37.72 |
| Lung | 28.5% | 0.24 | 28.04, 29.01 |
| Pancreas | 33.3% | 0.37 | 32.57, 34.03 |
| Other | 32.4% | 0.18 | 32.10, 32.81 |

Table S3. Average annual percent change (AAPC) in the proportion of death out-of-hospital by category (solid vs. hematologic malignancies)

| Cancer category | Period | AAPC | AAPC 95% CI (lower, upper) | *p*-value |
| --- | --- | --- | --- | --- |
| Solid | 1998-2018 | 1.2 | 0.6, 1.9 | <0.001 |
| Hematologic | 1998-2018 | 1.5 | -0.4, 3.3 | 0.124 |

AAPC - average annual percent change

Table S4. Trends in out-of-hospital cancer deaths, by district of resident, Israel, 1998-2018

| District | Segment period | APC | APC 95% CI (lower, upper) | *p*-Value |
| --- | --- | --- | --- | --- |
| Southern | 1998-2004 | 4.4 | 0.8, 8.2 | 0.02 |
| Southern | 2004-2018 | 0.5 | -0.3, 1.3 | 0.207 |
| Central | 1998-2003 | 5.6 | 1.2, 10.2 | 0.016 |
| Central | 2003-2009 | -2.3 | -6.1, 1.6 | 0.22 |
| Central | 2009-2018 | 4.8 | 3.4, 6.1 | <0.001 |
| Northern | 1998-2018 | -0.5 | -1.1, 0 | 0.055 |
| Haifa | 1998-2005 | 0.8 | -0.7, 2.4 | 0.264 |
| Haifa | 2005-2011 | -3.8 | -6.3, -1.2 | 0.008 |
| Haifa | 2011-2018 | 3.1 | 1.5, 4.8 | 0.001 |
| Jerusalem | 1998-2014 | -3.2 | -4.2, 2.3 | <0.001 |
| Jerusalem | 2014-2018 | 7.2 | -1.3, 16.4 | 0.092 |
| Tel Aviv | 1998-2018 | 2.3 | 1.9, 2.6 | <0.001 |

APC - annual percent change
