# Supplementary figures and images for: Place of Death for Israeli Cancer Patients Over a 20-Year Period: Reducing Hospital Deaths, but Barriers Remain
Source: Oncologist. 2023 Jun 1;28(11):e1092–8. doi: 10.1093/oncolo/oyad141 (PMC10628558; doi:10.1093/oncolo/oyad141)

Figure S1. Out-of-hospital cancer deaths by age and sex in Israel 1998-2018
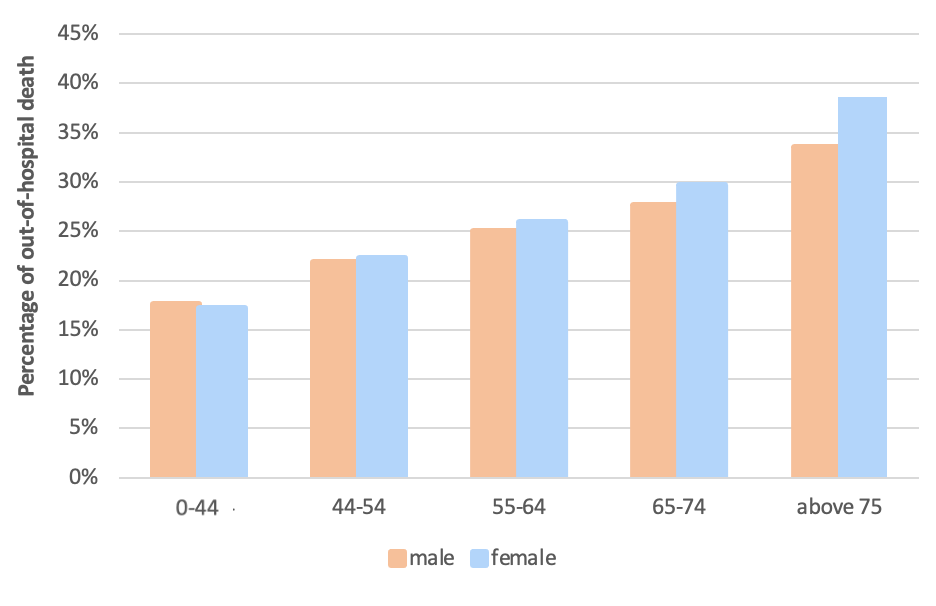

Supplement: oyad141_suppl_Supplementary_Figure_1 [file oyad141_suppl_supplementary_figure_1.docx]
